# Development and Validation of a Mobile Game for Culturally Sensitive Child Sexual Abuse Prevention Education in Tanzania: Mixed Methods Study

**DOI:** 10.2196/30350

**Published:** 2021-11-08

**Authors:** Maria Proches Malamsha, Elingarami Sauli, Edith Talina Luhanga

**Affiliations:** 1 School of Computation and Communication Science and Engineering Nelson Mandela African Institution of Science and Technology Arusha United Republic of Tanzania; 2 School of Life Science and Bioengineering Nelson Mandela African Institution of Science and Technology Arusha United Republic of Tanzania; 3 Carnegie Mellon University Africa Kigali Rwanda

**Keywords:** child sexual abuse, social cultural belief, ecological setting, prevention, parents, caretakers, child experts, mobile game

## Abstract

**Background:**

Globally, 3 out of 20 children experience sexual abuse before the age of 18 years. Educating children about sexual abuse and prevention is an evidence-based strategy that is recommended for ending child sexual abuse. Digital games are increasingly being used to influence healthy behaviors in children and could be an efficient and friendly approach to educating children about sexual abuse prevention. However, little is known on the best way to develop a culturally sensitive game that targets children in Africa—where sexual education is still taboo—that would be engaging, effective, and acceptable to parents and caretakers.

**Objective:**

This study aimed to develop a socioculturally appropriate, mobile-based game for educating young children (<5 years) and parents and caretakers in Tanzania on sexual abuse prevention.

**Methods:**

HappyToto children’s game was co-designed with 111 parents and caretakers (females: n=58, 52.3%; male: n=53, 47.7%) of children below 18 years of age and 24 child experts in Tanzania through surveys and focus group discussions conducted from March 2020 to April 2020. From these, we derived an overview of topics, sociocultural practices, social environment, and game interface designs that should be considered when designing child sexual abuse prevention (CSAP) education interventions. We also conducted paper prototyping and storyboarding sessions for the game’s interface, storylines, and options. To validate the application’s prototype, 32 parents (females: n=18, 56%; males: n=14, 44%) of children aged 3-5 years and 5 children (females: n=2, 40%; males: n=3, 60%) of the same age group played the game for half an hour on average. The parents undertook a pre-post intervention assessment on confidence and ability to engage in CSAP education conversations, as well as exit surveys on the usability and sociocultural acceptability of the game, while children were quizzed on the topics covered and their enjoyment of the game.

**Results:**

Parents and caregivers showed interest in the developed game during the conducted surveys, and each parent on average navigated through all the parts of the game. The confidence level of parents in talking about CSAP increased from an average of 3.56 (neutral) before using the game to 4.9 (confident) after using the game. The ability scores, calculated based on a range of topics included in CSAP education talks with children, also increased from 5.67 (out of 10) to 8.8 (out of 10) after the game was played. Both confidence level and ability scores were statistically significant (*P*<.001). All 5 children were interested in the game and enjoyed the game-provided activities.

**Conclusions:**

The HappyToto game can thus be an effective technology-based intervention for improving the knowledge and skills of parents and children in CSAP education.

## Introduction

### Background

Child sexual abuse (CSA) is increasingly becoming a serious public health issue affecting both boys and girls. It has been associated with physical, mental, and behavioral health problems that may be long lasting throughout the course of a child's life [1]. According to global evaluations and meta-analyses, the rates of CSA are estimated to be between 7.6% and 7.9% for boys and 18.0% to 19.7% for girls [2,3]. According to studies conducted in Uganda, India, Israel, and the United States, the COVID-19 pandemic has resulted in an upsurge in CSA [4-6]. In Tanzania, almost 3 out of every 10 females and 1 out of every 7 males experience some form of sexual harassment before the age of 18 [7]. Records from Tanzania’s Ministry of Health, Community Development, Gender, Elderly and Children (MoHCDGEC) show that the numbers have fluctuated regularly but have consistently increased: from 4423 cases in 2016 to 4307 in 2017 and from 9408 cases in 2018 to 10,750 cases in 2019. Such a trend is worrying and demands a whole-society approach in solving CSA. In most cases globally, the perpetrator has a close relationship with the abused child (eg, they are a parent, friend, or dating partner) [8]. Likewise, in Tanzania, the perpetrator is usually someone familiar to the child, such as a neighbor, teacher, dating partner, or relative (uncle or cousin) [7,9].

When children are sexually abused, it affects their physical, mental, and psychological health. There have been reports of both short-term effects, such as pain, bleeding, and harm to private parts, including tearing and even discharge; and long-term effects, such as psychological abuse, emotional torture, low academic performance, becoming sexual offenders, depression, anxiety, infertility in girls, and the acquisition of chronic diseases such as HIV/AIDS [1]. In recognition of these serious effects of sexual violence and exploitation of children, the United Nations sustainable development goals include a target (Target 16.2) to “end abuse, exploitation, trafficking and all forms of violence against and torture of children.” The World Health Organization, in partnership with various partners, also developed INSPIRE, a set of 7 evidence-based strategies that can be used by communities and countries to eliminate various forms of violence against children [10]. Among the strategies recommended is educating children about sexual abuse and its prevention.

CSAP education (CSAPE) is not a new concept. School-based programs have been adapted into the education systems of many countries, and they have proven to be very effective in transmitting information to children [11,12]. In Ireland for example, children aged between 6 and 12 years old are educated on topics such as safe and unsafe touches, not keeping secrets from parents, and the danger of strangers [13]. In North America, the United Kingdom, Australia, and New Zealand, school children are taught sexual abuse recognition, appropriate and inappropriate touches, the difference between good and bad secrets, how to say no and avoid unwanted advances, how to report abuse to an adult, and how abuse is never the fault of a child [12,14]. In Tanzania, no specific programs exist in the school curriculum to teach children about sexual abuse prevention [1]. However, schools may implement their own programs if needed. Unfortunately, organizing professional visits for students specifically for CSAPE is costly in terms of time and money and may therefore be out of reach for the children enrolled in primary schools, where scarcity of resources sometimes means even essential items, such as classrooms and desks, are unavailable [11,12,15].

Apart from school-based programs, CSAPE can also be conducted through places of worship and media campaigns. In Tanzania, a the 2019-2020 report by the MoHCDGEC highlighted that churches, mosques, books, and campaigns from various ministries and government agencies, among others, had offered CSAPE programs in the 2019-2020 period to 72,832 children. Similarly, in the United States, a community preventative technique that includes home visits and technological enhancement tools (eg, mobile phones, websites, phoning, SMS text messaging) to educate parents on positive parenting has shown encouraging results [16-18].

A key limitation of school and other public-based programs is that younger children are not yet in school. Younger children who are not yet in school (usually under 6 years old) are not considered in the development of the CSAPE programs. Yet, sexual abuse among this age group is present and can be perpetuated by myths, such as the belief that HIV infection can be cured through sexual contact with a child [9]. The lesser-developed communication skills of this age group may also mean that they are unable to report incidents of abuse. Home-based CSAPE is thus important to ensure their safety. From the age of 1 to 6 years, children grow sexually, recognize their biological differences, exhibit sexual gestures, and become explorative [19]. Children between 3 and 6 years look to their parents and caretakers for answers to questions such as the difference between girls and boys. Studies have proposed that parents should create home environments where they are engaged and ready to respond to and openly discuss these kinds of topics [20-23]. Child psychologists recommend that talks on sexual abuse with this age group should focus on the same topics offered to older children, including those regarding private parts, safe and unsafe touches, the importance of not keeping secrets from parents and caregivers, requesting permission before going anywhere, and reporting situations where they did not feel safe, and that these talks be offered in an easier language [24]. For many parents, this can be a daunting task. In Tanzania, there are additional sociocultural barriers, such as the discussion of sex being generally taboo, especially that occurring between parents and caretakers and their children [25].

Digital games have increasingly been used to offer health interventions to children and their parents. Mobile learning games are games created with the defined goal of deploying on a mobile phone to motivate and engage society, especially children, on a predetermined topic [26]. Mobile learning games provide learning anywhere and anytime, and allow children to take more risks [27] and present the opportunity to retry following failure [26]. Mobile learning games geared toward health include eBug and modified Mario Brothers games. Mobile learning games specifically targeting sexual abuse prevention include Cool and Safe, Orbit, and SAP MobAPP. With the rising mobile and internet use penetration in Tanzania (29 million citizens in June 2020), mobile learning games can be a useful resource for providing CSAPE to parents and their younger children. However, none of these games have been developed with the African sociocultural context in mind. For instance, body parts like the buttocks are referred to using their proper names, which is widely unacceptable in Africa, and extended families are not featured. Their general acceptability and effectiveness may therefore be hindered.

### Objectives

This study aimed to develop a CSAPE mobile learning game to target the 3 to 5-year-old group, tailored for Tanzania as a case study. The acceptance of CSAPE game is associated with cultural relevance [28] and co-designed with users of digital apps. Therefore, the game was designed with a mixed methods approach to identify parents’ and caretakers’ requirements, as well as with child experts’ recommendations for the game content and design [29]. The following research questions were thus examined: What are the parents’, caretakers’, and child experts’ topics and content related to CSAPE that should be contained in the game? What are the challenges related to parents’, caretakers’, and child experts' sociocultural and environmental condition that should be considered in the CSAPE in Tanzania? What are the parents’, caretakers, and child experts’ requirements for the game interface, features, and interaction designs? What would the acceptability and usability of a CSAPE mobile app be to society?

### Related Work

Examples of computer-aided sexual abuse prevention games for children include Cool and Safe [30], which is an effective web-based prevention training program containing film clips, stories, tasks, and games developed for French and German elementary school children. Safety for Kids 3 is another mobile animation app to teach children about safety in their environment, including preventing CSA [31]. Other educational games include Orbit, which is Australian web-based software for children aged 8 to 10 years that offers information on togetherness, listening, understanding, belief, and courage [27]. There is also SAP MobAPP, a mobile app for primary school children in Korea [28]; and the Child Sexual Abuse Prevention Education Using Hybrid Application, which includes a combination of a website and a mobile app as a behavioral self-protection tool for fifth graders in South Korea [32]. Stewards of Children is also another combination of website and mobile app used for raising awareness on the prevention, recognition, and reaction to CSA using real people’s stories [33].

In designing game-based learning, cognitive, affective, motivational, and sociocultural theoretical foundations play a significant role [26]. The cognitive foundation suggests that all the tasks and activities in the game should reflect the lessons to be learned. The motivation foundation emphasizes the need for the game to have activities that will interest and engage a player in the tasks. Moreover, the affective foundation explains how features of a game make a player feel, thus affecting their learning (eg, warm colors that may attract a child). Lastly, social and cultural foundations have a role to play in game design by connecting it to the player for easy learning. Understanding a community's sociocultural practices can significantly contribute toward a reliable and appropriate prevention program. Certain traits exist in Tanzanian communities that should be considered when developing a prevention program, such as family structures, sex and gender taboos (modesty, gender roles), sexual norms, separation (male and female), the culture of silence, foreign influence, religious teachings, unquestionable obedience by children, the value of children, poverty, and lack of explicitness in teachings [34]. As a result, any preventive measures must consider the child’s social environment (working together with the entire environment to which a child is exposed), and the appropriateness of any such approach must include parents [35]. The design must also consider children's engagement in the game (flow theory: balancing between boredom and anxiety) [36]. This flow includes the magic circle of game-based learning, which involves response, feedback, and challenge [26].

## Methods

A preliminary survey was conducted in 3 regions of Tanzania: Dar es Salaam in Ubungo district, Arusha in Meru district, and Morogoro in Mahenge district, along with other regions with a very small percentage of participation. The regions were selected to provide the sociocultural and social setting requirements for game development. Dar es Salaam was selected because of the presence of multicultural interactions in an urban setting, Morogoro was chosen because of multicultural interactions in a rural setting. and validation was done in Arusha where there is also multicultural interactions in an urban setting. The first phase of the survey, completed in Dar es Salaam, Morogoro, and other areas, involved questionnaire and focus group discussion from March 2020 to April 2020. The second phase of the survey was completed for validation and was conducted in Arusha from March 2020 to April 2020.

### Sample Size

A total of 172 people participated in the study. In the first phase, 111 parents from various districts (Ubungo, Ulanga, Meru, and others) were involved in the study survey, including 58 females (52.3%) and 53 males (47.7%). Twenty four child experts forming four focus groups from two districts (Ubungo and Ulanga) took part in discussion from March 2020 to April 2020 to design the game content, features, interface, and interactions. In the second phase, 32 parents and 5 children from Meru district participated in the validation. Participants were given information about the facts and benefits of the research before they agreed to participate.

### Sampling and Recruitment

To obtain a representative sample of the population in the first phase survey, a snowball sampling technique was used [37]. Parents were selected from strategic locations: outside of their children's schools, hospitals, market places, home visits. and online Google forms via email contact lists or WhatsApp groups for answering questionnaires. Participants were asked if they would be willing to recommend another person who fulfilled the criteria, was willing to take part, and lived nearby. Similarly to the online questionnaire, participants were requested to forward it to others who met the criteria. We recognized that participation in the study may have unintended implications, such as the likelihood of emotional distress for persons who have experienced sexual abuse. To minimize this, both the introduction and the form of consent stated that the nature of questions was focused solely on app design and not on experiences. The survey questions were reviewed and approved by Kibong’oto Infectious Diseases Hospital-Nelson Mandela African Institution of Science and Technology-Centre for Educational Development in Health, Arusha Health Research Ethics Committee. The ability to exit the survey at any time was emphasized. Child experts were selected from the local government gender desks to district social service offices. One or two hosts were identified and informed about the study, and they recommended which experts should be included in the focus group discussion. During validation in the second-phase survey, parents were sampled using the snowballing technique during home and office visits.

### Inclusion Criteria

To be included in the study, participants had to be 18 years of age or older, own a smartphone, and have at least 1 child aged 1 to 18 years. Both male and female parents and caretakers were eligible. To be regarded as a child expert, the individual had to possess the necessary abilities and knowledge to protect children's safety and well-being. These participants completed a first-stage survey for the requirement selection.

To be included in the second-phase survey, participants had to be 18 years of age or older, own a smartphone, and have at least 1 child aged 3 to 5 years. Both male and female parents and caretakers were eligible. Before participating in the survey, participants were required to sign a consent form after they were informed about the study.

### Parents and Caretakers Requirements

For data collection in the first survey, which was conducted face to face and online, a paper questionnaire with 33 questions separated into 4 sections was prepared (Multimedia Appendix 1). There were different types of questions, including dichotomous-response (yes or no), multiple-choice, and open-ended questions. Section 1 collected demographics, such as age, gender, marital status, number of children, and educational background. No personal information was collected, but parents and caretakers who agreed to take part in additional research phases were asked to provide their names and contact information separately after completing the survey. Parents and caretakers were free to provide or not provide such information. Section 2 included questions aimed to determine current CSAPE practices in Tanzania, such as parents’ skills, knowledge, the child’s age at which they are comfortable for their children to begin learning CSA prevention education, and challenges and enablers of parents talking to their children about CSAPE. Game specifications, mobile games, general games that children enjoy playing, and whether or not parents allow their children to use their phones were all discussed for Tanzanian children in Section 3. This section also featured elements that encourage a child to play a specific game, as well as play mode preferences for parents or caretakers and their children. The draw-write-tell method was used in this section to allow parents and caregivers to contribute in the design of the gamed user interface (UI) [38]. Section 4 inquired about the social environment factors that contribute to CSA in Tanzanian families, such as family structures and how often parents examine their children for symptoms of abuse.

### Child Expert Requirements

The purpose of the focus groups discussion was to try to understand the challenges for parents and caretakers in talking to their children about CSAP, topics to include in CSAPE, and how the game should be designed (Multimedia Appendix 2). In each session, the facilitator introduced the purpose, and then participants could discuss them while the facilitator made sure everyone was heard. Interviews with child care experts were then conducted. The interviews were semistructured with open-ended questions and split into 4 sections. Section 1 consisted of demographic information. Section 2 comprised questions on the existing policies and methods used to protect children, topics and sociocultural requirements for prevention education against child sexual exploitation, and the obstacles that come with it in Tanzania. Section 3 asked about the social environment that contributes to child sexual abuse. Section 4 was about game UI design and using the draw-write-tell method to explain their design ideas [38]. To promote co-design with the mobile users, we also conducted paper prototyping and storyboarding sessions for the game’s interface, storylines, and options in focus groups discussions with child experts.

### Game Development Approach

Transforming the user’s requirements into a software platform is referred to as development process. In this study, the game development life cycle (GDLC) model [39] was used as the development process. The GDLC involves preproduction, production, and postproduction phases. The benefit of GDLC is that it can handle the multidisciplinary nature of game development, which includes a mix of art, sound, control systems, human factors, and artificial intelligence to form a creative concept in order to entertain and teach, as opposed to conventional software development which aims at solving problems [40,41]. Due to time and budget constraints, the scope of the app was limited to include content for 3 to 5-year-old children and parents acting as a guiding presence.

The preproduction phase involved the requirements data collection. After data analysis, game design and game prototype were used to put together a comprehensive design document detailing the game goals, storyline, fun factors, level designs, gameplay mechanics, and overall blueprint of the game [40]. The production phase consisted of the development of different game components including storyboard production, assets creation, development platform, game engine, programming, implementation of HappyToto, and gameplay. The evolutionary prototyping approach was used, where the first version is created after obtaining input from the users, and then subsequent versions are made with additional functionality. The tools and technologies used in development of the game included a flutter platform that provided the design for the game by combining artwork, graphics, sound, code, and engine. It is a single code-based platform app that can be used to build software for both Android and iOS devices [42]. Dart, a Google-developed object programming language, was also used to develop the game. Dart focuses on front-end creation for mobile and web applications. Additionally, Adobe Illustrator software was used to create and draw 2D vector graphic objects, while Adobe After Effect was used to develop the motion graphics and visual effects of the game. Sound was recorded by sound recorder software. The postproduction phase consisted of game beta testing and validation. The researcher and 2 parents tested HappyToto, after which development and errors were rectified.

### Game Validation

Validation for this HappyToto game took place between February 2021 and March 2021. The application prototype was validated using a survey with 32 parents of children aged between 3 and 5 years, including 18 females (56%) and 14 males (44%); and with 5 children, including 2 girls (40%) and 3 boys (60%) of the same age group. A pre- and postintervention assessment was used to determine the comfort of parents in allowing their children to use HappyToto as an education toolkit for CSAPE, parents’ usability of the game, and children’s’ perception of the game (Multimedia Appendix 3). Questionnaires, interviews, and observations were used for both intervention assessments. The questionnaire was used to ask parents about their comfort in talking to their children about CSAP before using the game. The parents were then allowed to engage with the game while being led by the researcher. Noninterruptive data were collected based on observation as the user interacted with the system, and all the comments made by users while using the game were noted. Parents were asked to score their confidence in talking to their children about CSA after playing the game, and usability questions were asked. Open-ended questions were also asked, such as their favorite feature, the least-liked feature, and what could be added or improved to make the game more appealing and appropriate. The data collected from children included how well they could follow instructions and navigate through the game and their interest in levels while playing.

### Data Analysis

Questionnaires, interviews, and observations were used to collect both qualitative and quantitative data. For qualitative data, the inductive method for qualitative analysis was used. For quantitative data, descriptive statistics (frequency) were used to investigate the data while *t* tests were conducted using R software (The Foundation for Statistical Computing) [43] to detect any significance difference between the ranks of CSAPE description from parents before and after using the game.

### Ethical Considerations

Before taking part in the study, the qualified participants were informed of their rights, free will to participate, the type of research, what data were needed, how their data would be published in aggregate, and that there would be no adverse impact whether they chose to participate or not. Participation was limited to the parents and child experts whose informed consent was signed, while for the children, this involved a trusted adult. The HappyToto game development study received ethical clearance for each stage of its development and validation from Kibong’oto Infectious Diseases Hospital-Nelson Mandela African Institution of Science and Technology-Centre for Educational Development in Health, Arusha Health Research Ethics Committee.

## Results

### Parent, Caretaker, and Child Expert Requirements

A total of 111 parents and caretakers (females: 58, 52.3%; male: 53, 47.7%) were distributed across the regions of Arusha (n=11, 9.9%), Dar es Salaam (n=54, 48.7%), Morogoro (n=20, 18%), and others (Dodoma, Iringa, Kagera, Kigoma, Kilimanjaro, Manyara, Mbeya, Mtwara, Mwanza, Pwani, Shinyanga, Songwe, and Zanzibar: 26, 23.4%) during phase 1 of the survey. Interviews involved 24 participants from Dar es Salaam (n=19, 79%) and Morogoro (n=5, 21%) and included 18 (75%) females and 6 males (25%) as indicated in Table 1.

**Table 1:** Socioeconomic and demographic characteristics of participant parents, caretakers, and experts.

Characteristic			Phase 1 (requirement gathering)		Phase 2 interview (validation), n (%) (N=32)
			Survey, n (%) (N=111)	Interview, n (%) (N=24)	
**Regions reached**					
	Dar-es Salaam		54 (48.7)	19 (79)	0 (0)
	Morogoro		20 (18)	5 (21)	0 (0)
	Arusha		11 (9.9)	0 (0)	32 (100)
	Others^a^		26 (23.4)	0 (0)	0 (0)
**Gender identity**					
	Women		58 (52.3)	18 (75)	18 (56)
	Men		53 (47.7)	6 (25)	14 (44)
**Age (years)**					
	20 -30		43 (38.7)	4 (17)	14 (44)
	31- 40		40 (36)	15 (63)	14 (44)
	41 and above		28 (25.2)	6 (21)	4 (12)
**Education**					
	Primary school		9 (8.1)	3 (13)	10 (31)
	Secondary school		12 (10.8)	5 (21)	10 (31)
	Tertiary level		81.1 (90)	16 (67)	12 (38)
**Employment**					
	**Employed**		70 (63.1)	N/A^b^	21 (66)
		Social officer	N/A	19 (79)	N/A
		Teacher	N/A	3 (13)	N/A
		Police officer	N/A	1 (4)	N/A
		House maid	N/A	1 (4)	N/A
	Self-employed		35 (31.5)	N/A	10 (31)
	Farmer		18 (5.4)	N/A	1 (3)
**Number of children**					
	1		43 (38.7)	N/A	12 (38)
	2		27 (24.3)	N/A	11 (34)
	3		17 (15.3)	N/A	5 (16)
	4		9 (8.1)	N/A	3 (9)
	5 and above		15 (13.5)	N/A	1 (3)

^a^Including Dodoma, Iringa, Kagera, Kigoma, Kilimanjaro, Manyara, Mbeya, Mtwara, Mwanza, Pwani, Shinyanga, Songwe, Zanzibar.

^b^N/A: not applicable.

#### Topic Suggestion

The main topics which parents, caretakers, and child experts were interested in were dangers of receiving presents (83/111, 74.8%), modesty and body changes during different stages of growth and development (71/111/, 64.4%), what is an abuse and who can be an abuser (70/111, 63.0%), dangerous people and environments and what to do if encountered (63/111, 57.0%), safe and healthy versus unsafe and unhealthy touches (60/111, 54.1%), abandoning bad traditions (43/111, 38.5%), private parts names (32/111, 28.9%), fear of God (16/111, 14.8%) and finally confidence (12/111, 11.1%). The locations that parents, caretakers, and child experts felt children spent the most time (social environment criteria) and could be vulnerable to abuse were the “home,” “school,” “on the way to/from school,” “playing outside with friends,” and “in religious institutions.” As a result, they felt the game should discuss the recommended topics in these settings (eg, receiving gifts at school, on the way home).

#### Sociocultural Requirement

Around 70 of the 111 parents, caretakers, and child experts (63.1%) feared that exposure to CSAPE would affect children’s innocence and thus potentially make them more sexually active. Other concerns highlighted by parents, caretakers, and child experts were that they did not have enough skills to teach their children (46/111, 41.5%) and that Tanzanian society did not favor openness to children (40/111, 39.3%) and therefore felt it prudent to not expose children to taboo subjects. The other fear included busy schedules (21/111, 19.3%), globalization (16/111, 14.1%), the CSA threat not being that great (13/111, 11.9%), poverty (11/111, 9.6%), and increase of existing temptations from society (10/111 8.9%).

#### UI Design From Parents, Caretakers, and Child Experts

Parents, caretakers, and child experts designed their ideas for the game in the first phase of the survey by drawing, writing, and telling [38] what they wanted to see as the game's UI. The UI sketches and explanations are used as guidance when designing the game's UI and include layout and environment design. Parents, caretakers, and child experts also preferred the Tanzanian setting, characters, and objects surrounding the children; use of Swahili language; and the and less explicit content. Furthermore, a preference survey for operating systems was conducted, with Android (Google; 93/111, 83.8%) and iOS (Apple; 9/111, 14.4%) being the most preferred.

### HappyToto Game Design and Development

**Figure 1:**
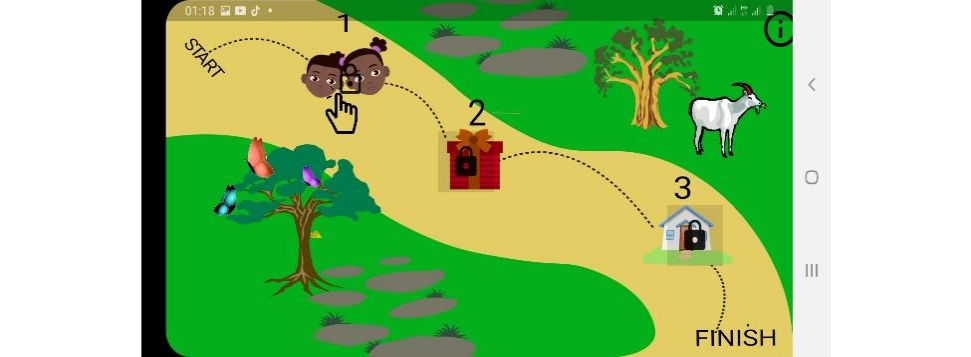
Game landing page (levels page).

HappyToto is the name given to the mobile game developed for 3 to 5-year-old children. The story script was composed in Swahili and English languages with consideration to the parents’, caretakers’ and child experts' opinions from the surveys and the stipulated child prevention education from previous studies. The game consists of 3 levels: private parts, presents or gifts, and a safe environment (see Figure 1).

#### Storyline and Storyboard Production

The storyline is one developed based on today's Tanzania where Ibra and Elly are twins from a middle-class household. As they are playing outside, their mother teaches them different lessons related to private parts. On the second level, their mother teaches them about proper presents to receive in the flipping game and about not receiving presents from unfamiliar people. After remembering the lessons their mother taught them, the story ends with the mother teaching Ibra and Elly about safe places and safe people inside the house and how they behave outside their home. Following each lesson, there is an exercise to check understanding. If the exercise is passed, the level is complete.

HappyToto contains 2 main cartoon-type characters Ibra (a boy) and Elly (a girl) with body shape, facial features, hair, brown skin, and clothes that reflect Tanzanian children that are 5 years old. Tanzanian children like to play and to listen to their parents. Other characters include a mother who is not physically present in the game but whose orders are narrated, a guest, and a stranger.

**Figure 2:**
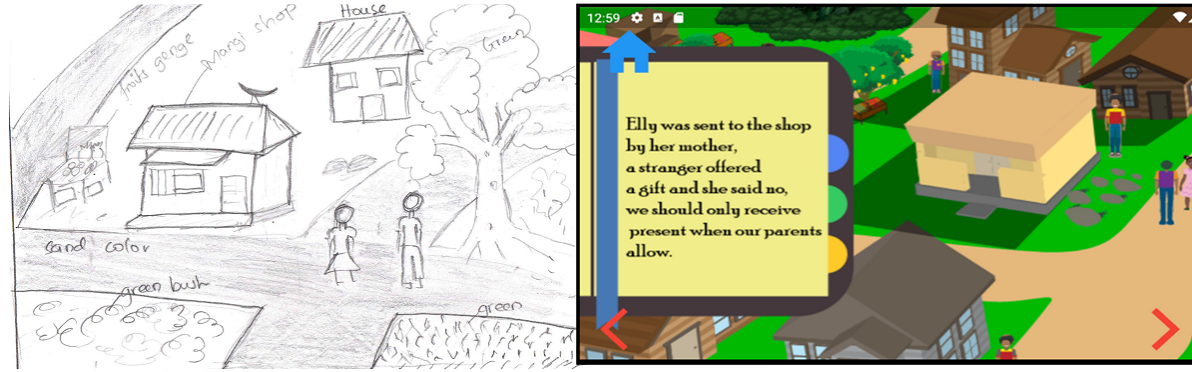
Sketch of the outdoor environment and 2D of the outdoor environment.

Game scenarios were sketched with paper and pencil for each setup in the levels, character, environment, and story flow for different scenes to depict Tanzanian settings. During the phase 1 survey, parents, caretakers, and child experts participated in the paper and pencil design of the game's UI (see Figure 2).

#### Assets Creation

The game assets included audio, interface design, and icons that are stored within the app. Background audio was obtained from open-source libraries, and narratives, instructions, and applause were recorded using a voice recorder application. The framework provided the user interface widgets, including icons for the next, back, and home buttons. The other user interface resources included images, video, buttons, and pop-up notifications. The environment and characters were created as 2D vector graphics in Adobe Illustrator using child-friendly colors like red, blue, and yellow. Adobe After Effect was used to create the game's video segment, including merging 2D objects and storylines and animating them.

#### App Screens

HappyToto consists of 2 welcome screens, where the first contains information about the game and the next contains the levels of the game. There is also an information button at the top right for adults in case they want more resources on CSA prevention, and access requires users to solve a mathematical problem. Level 1 consists of 4 screens: private parts, safe touch, dressing, and self-care. Level 2 consists of 4 screens: presents, flipping, presents outdoors, and dragging. Level 3 consists of 7 screens: safe places outdoors, safe places in the market, safe places indoors, safe places in the house, who to ask for help, safe people, and reactions when someone makes you sad.

#### Gameplay

When the level 1 button is pressed, a video on private parts with a play button instructs the player to play. The video tells the tale of what private parts are, and the next screen contains a safe touch exercise. The next screen contains a dressing game in which a child must drag clothes to the 2 characters. The final screen of level 1 includes 2 clickable characters that, when pressed, provide a narrative of what activities Ibra and Elly may do on their own and whom to ask for assistance. When the next button is pressed it gives instructions to activate the second round. When a child lands on level 2, narratives about receiving presents begin, and the following screen features a flipping card game that shows examples of gifts that a child might receive. The subsequent screen narrative focuses on the dangerous places to receive presents and is followed by an image-dragging exercise in which after a picture is completed, after which a lesson on what a child should be mindful of is given. The level ends with instructions on the landing page on level 3, which is subsequently unlocked. Level 3 starts with a narrative on a safe environment outdoors, followed by hide and seek as an exercise to locate safe and unsafe places. The fourth page in this level starts with a narrative on a safe environment indoors, followed by a hide-and-seek exercise to locate safe and unsafe places and people in the house. The end screen of the game describes what Ibra and his friends should do upon encountering dangerous people. The game has a flow mode storyline which follows the same children across different days, as this was the favored mode from the survey. There is also looping music played at each level to continue engaging a child.

### Game Validation Results

Thirty-two parents in the Arusha Region participated in the phase 2 survey for validation. Of the participants, 44% (14/32) were men, 56% (18/32) were women, their ages ranged from 20 to 50 years, 31% (10/32) had received primary education, 31% (10/32) had received secondary education, and 38% (n=12/32) had received tertiary education (Table 2). Children who participated in the evaluation were between 3 and 5 years old, with 60% (3/5) being male and 40% (2/5) being female.

**Table 2:** Comparison scores for confidence level and ranked results before and after using the game.

Education level		Confidence			Ranked results^a^		
		Topic 1	Topic 2	Topic 3	Topic 1	Topic 2	Topic 3
**Primary level**							
	Pretest mean	3.20	4.20	4.3	5	5.6	5.8
	Posttest mean	4.90	4.9	5.00	9	8.9	8.8
**Secondary level**							
	Pretest mean	3.7	4.2	4.2	6.3	6.6	6.6
	Posttest mean	5	5	5	9.2	9.2	9.2
**Tertiary level**							
	Pretest mean	2.58	3.08	2.83	4.75	5.16	5.25
	Posttest mean	4.83	4.83	4.92	8.38	8.29	8.38

^a^Two-tailed (*T* ≤ *t*) *P* value <.001.

#### Game Acceptability

Parents were generally happy with the game; out of 32 interviewed, 81% (n=26) scored as a “satisfied parent” (awarding it a 5 out of 5), while a score of 4 out of 5 (“somewhat satisfied”) was given by 19% (n=6), as shown in Table 2. Two of the participants (T3 and T2) requested permission to download the game to their phones immediately. One hundred percent of the participants (n=32) would allow their children to play the game immediately if they were around and said they would recommend this game to other parents. On the features generally, 90% (n=29) of the parents liked game level 1, 81% (n=26) liked game level 2, and 78% (n=25) liked game level 3. The reasons for parents’ preference for all the levels were the way the lessons were well integrated into the game activities (15/32, 50%). The safe touch activity, dragging, story narration, and visuals used were all liked by parents. The storyline for teaching sensitive topics in a creative and appropriate way and the in-game activities were praised by all participants (n=32). For example, participant S8 said, “I love how the lessons incorporated in hiding and seek feature because children can relate with it,” and T11 commented, “It is very educative to children especially the private part lesson and the dragging features.”

The private parts topic was completely acceptable because “colloquial words used” and the names of private parts were not called explicitly, respectful and motherly language was used, and the game conformed to Tanzanian culture, with 100% (n=32) of the participants rating it 5 out of 5 (“appropriate”). Also, 94% (n=30) of the participants ranked the “receiving presents” subject 5 out of 5 (“appropriate”) and the “dangerous environment and people” subject was ranked 5 out of 5 (“appropriate”) by 84% (n=27) of all participants. The language (Swahili), not explicitly naming private parts, characters and climate, indoor and outdoor settings, and family structures were the basic features listed as conforming to the culture.

In general, the participants discussed some features including respectful, friendly, and nonexplicit language; age-appropriate lessons; inclusion of local games such as “kombolela” (hide and seek); and graphics of Tanzanian characters and environments as very useful for displaying the UI.

Although they were satisfied, when asked “What is your least favorite feature?” and “What features should be added/modified,” 28% (n=9) of participants indicated technical corrections such as the need for more instructions, icons, animation, and game analytics. For instance, participant T4 said, “I wish there were more animation in the game and more restrictions in the level where a child will play for some time before going to another level”; T6 said, “You can add game analytics to allow parents so see the progress of their children and what features they enjoy and play a lot.” Twenty-eight percent (n=9) of the participants recommended modification to the story by adding more scenes and adding detail on how to talk to children. Participants T10 and P3 suggested to “add more dangerous environment and dangerous people scenes” and “add scenes where the emphasis is put on a child to report to a parent how their day went,” respectively. Nineteen percent (n=6) recommended variation of a narrator sound incorporating a man’s and child’s voice with strictness in the unacceptable behavior. Participant P4 suggested, “You need to add more lessons related songs to make the game more fun.”

When asked about the possible effects of the game, 84% (n=27) of the participants rated it a 1 out of 5 (“not affected”). This was because the game was engaging but not to the point of addiction, and because it is offline, children cannot reach other sites. The remaining 16% (n=5) of participants were concerned about the effects of light from the mobile phone as well as addiction to the game.

#### Pre- and Postconfidence and Ability Scores

Before and after using the app, the trust level in educating children about private parts, obtaining gifts, and unsafe environments of people was measured using a 5-point Likert scale ranging from 1 (uncomfortable) to 5 (comfortable), as shown in Table 2.

The ranked results were obtained when parents were asked objectively how they intended to educate a child, and the results were ranked by taking into account the key points in each topic and guidelines from MoHCDGECs National Parenting Education Manual for Families. The parents’ opinions of the game before and after using it are summarized in Table 3. Prior to interacting with the game, parents had different views on the topics. Some parents assumed that their children were still too young (3 years) and that they would not understand until they were approximately 5 years old or teenagers. Some parents spoke with their children in order to protect them because occurrences on the streets had given the impression that their children were not resistant to violence. Of the 32 parents who were asked whether they were familiar with the cartoons that their children enjoyed watching and playing, 30 parents indicated having seen popular Swahili language children’s cartoons (eg, *Akili and Me* and *Ubongo Kids*).

**Table 3:** Selected parents opinion before and after using the game.

Participant characteristic		Exemplar quote
**Attitude before playing the game**		
	31 to 40-year-old woman	“I know there are effects, but I do not want to be very open to the child unless they ask; I am a doctor, so I know what to do and the effects. I do normal disciplining and emphasize a safe environment and people.”
	41 to 50-year-old man	“I provide child self-protection like no touch of the pupu [anus] and susu [vagina] parts; only receive presents from parents and no one else.”
	31-40-year man	“I do not talk to them about these issues; their mother does, and they are still young to be taught about sexual abuse.”
	41 to 50-year-old man	“They were not exposed to a dangerous environment, so there was no reason to tell them, but as their brother is approaching adolescence, he has started asking questions about his body changes.”
	41 to 50-year-old female	“I feel like my child is too young to understand all dangerous environments. He understands some, but not all the scenarios are easy to explain to him.”
	21 to 30-year-old female	“The neighboring child was abused, and there was a case I do not know how it ended; I have to scare my child so that she is not abused.”
	31 to 40-year-old female	“These abuses happen in town areas. My children live in the interior village, so they are very safe”.
**Overall acceptability**		
	31 to 40-year-old female	“The language is very well understood, attractive environment, lessons are well understood, and it matches with the age of the child.”
	31 to 40-year-old female	“The language used is not very explicit in naming the private parts; polite way.”
	41 to 50-year-old female	“This is very creative way of teaching.”
**Addiction potential**		
	20 to 30-year-old female	“For what I see this game, a child can’t not be addicted because there are no features to continuously engage a child. It is just for lesson and may be repeat a few times.”

#### Usability

**Figure 3:**
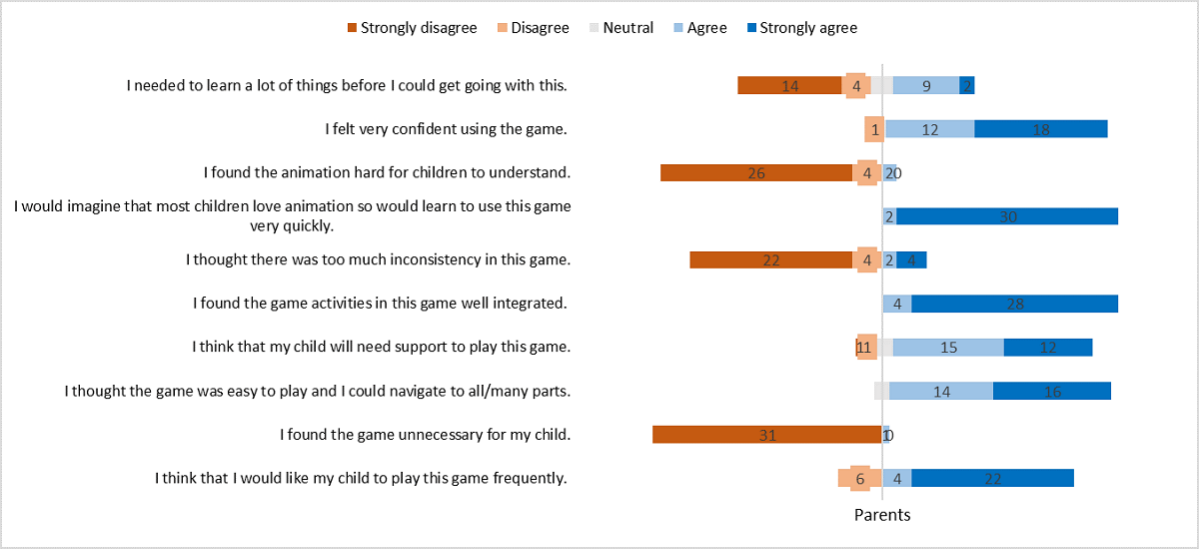
Usability test results.

Most parents felt confident using the game after reading the instructions. It was suggested that children will also need instructions during the first-time interacting with the game (see Figure 3).

#### Children Usability Results

All 5 children enjoyed flipping, dragging, and the ability to move around the game. When the level page loaded and they could hear the background sound, all the children became curious. After playing the game, the 4 to 5 year olds were able to correctly answer questions such as “What do you do if a neighbor tells you to take off your clothes?” A researcher met a 5-year-old girl after a day, and she showed an interest in playing the game again. They all expressed the need for more animations and activities or actions in the game, similar to what they had seen in movies to hold their attention while playing.

## Discussion

We have attempted to develop and validate a user-friendly children’s game app for prevention of sexual abuse. The distinguishing features for this study compared to other CSA prevention studies are the following: it is the first study in Tanzania to consider development and validation of the acceptability and usability of a game as a prevention education tool for children aged 3 to 5 years, Tanzania's culture, society, and social environment were considered in the design of the game, the content of the game touched on topics that parents and child experts indicated would be suitable, and a mobile game was used to improve child's learning experience without time or space limitations. Although other research has found significant results when using games [30], these studies considered other cultural contexts. CSAPE game assessment needs to be performed in Tanzania due to cultural differences and the increasing numbers of parents now using smartphones and allowing their children to play games on their phones. CSAPE games can provide another tool for creatively educating CSAP preschool children and keeping them engaged. We discovered that parents were afraid of taking away their children's innocence but also wanted them to learn about presents, body changes as they grow older, who an abuser is, reporting, and safe touches in a fun and culturally sensitive way. After the game was developed, parents and children were very interested in how the game was structured and showed gained skills. Herein, we discuss the usefulness and efficacy of the HappyToto game in CSA prevention.

All 32 parents were eager to introduce CSAPE to their 3 to 5 year old using HappyToto as a CSA prevention method because the lessons were age appropriate [19], but parents also believed that good parenting was the best way to protect children [20]. Proper parenting can be defined in 2 ways: (1) teaching children how to defend themselves (ie, teaching them to report to their parents when someone makes them feel uncomfortable) [20] and (2) limiting parenting for child safety, such as prohibiting children from going to specific locations where there is a high risk of seeing a perpetrator and imposing a curfew time to be back at home [20]. During the presurvey, each parent expressed an opinion about parenting in their personal situation. Without having seen a definition of parenting, each parent described parenting as one or both of the 2 definitions above. It is important to remember that even the notion of “proper parenting” is a transitory statement, as the definition of parenting has evolved as a result of the changing social environment and globalization. Most parents confessed to being aware of abuse but having difficulty discussing it openly with their young children [44] (ie, on the private parts of their bodies). Parents also expressed struggling to describe concepts such as “your uncle could abuse you.”

Previous research has shown that parents find it interesting how games can be used to teach prevention education and encourage their children to use them and agree that this idea should be used in the concept and design of programs and applications [45]; we therefore used this concept in this study and received positive feedback. It was surprising to see the parent, caretaker, and child expert participants’ contributions to CSA prevention tailored to a game, perhaps because they had not used such a game before. The role of a parent as a guiding presence while children play allows a parent to participate in CSA prevention [9] by providing additional clarification to children on concepts. HappyToto also fills the skills gap, where parents previously depended on skills learned as children [25], which were insufficient. HappyToto provides the skillset parents need to be more transparent and ready to overcome the taboo of not talking to children about issues of child sexual abuse. The confidence level of parents on talking about CSAPE with children significantly increased after using HappyToto (*P*<.001), as compared to before interacting with HappyToto, with the confidence level increasing from an average of 3.56 “neutral” before using the game to a 4.9 “confident” after using the game. Furthermore, when asked to describe how they would talk to their child before using the HappyToto game they scored an average of 5.67 out of 10 as compared to 8.8 out of 10 after using the game.

HappyToto offered several suggestions for talking to children about challenging topics in a nondirective manner that parents were willing to try. Due to its cultural appropriateness, the opportunity to connect to the game played an essential role in ensuring that it was acceptable to parents. Beginning with language (a woman in the game softly narrated in Swahili, Tanzania's official and national language), a safe environment was created for children to feel comfortable with the content [46]. Although CSAPE recommends that children be explicitly taught about healthy and improper interaction by giving proper names to private parts, Tanzanian culture's conservative nature makes it much more suitable to use colloquial words in the game [25,34]. The deliberate use of cartoons with brown skin color who are traditionally dressed provided commonly demanded values for society's children. The inclusion of fathers in the CSA prevention criteria debunked the idea that mothers were solely responsible for their children's education and gave fathers a chance to participate [47]. Parents who liked the game the least indicated that it was due to a lack of details in the lessons they would like to see and expressed the need for additional functionality.

In the first phase survey, parents with a secondary level education had an average of 3.9 (“somewhat confident“) while parents with a primary level education had an average of 4 (”somewhat confident“), indicating that they were more interested in educating their children about CSAPE than were parents with a tertiary level education, who had an average of 2.83 (“neutral”). This may possibly be due to the community lifestyle where the children are more likely to play in streets [45] and where people with a higher education are more likely to live in an area with fenced perimeters, where children generally do not move outside of their parent's view. It was okay, for example, for 3 to 5-year-old children who did not live inside the fences to receive a lesson in the game on how to behave on the walk to the shop because this is where sexual harassment occurs [9]. The fact that the game depicted real locations where abuse occurs was appealing to both parents and children, as it made the game relatable while still making the lessons easy to remember [28].

The storylike game with a narrative flow and basic activities were sufficiently fun for the children to play and help understand lessons while not being too addicting [46]. This feature increased parents' eagerness to encourage their children to play, knowing that they are still going to balance the game's use with other activities. Although the repetition function is necessary to ensure that children remember the material, this needs to be a balanced with the risk of addiction.

Parents were likely comfortable with the cartoon presentation and narration of HappyToto because the game was designed with the interests of preschoolers being considered [48]. The HappyToto design is also similar to culturally appropriate and relatable children educational cartoons in Swahili, such as *Akili and Me* and *Ubongo Children*. Suggested improvements from participants were to include variations in the voices of women, men, and children but also strictness in the voice when anything that should not be done was being discussed so that children can be properly informed [47].

Several limitations became evident in this study. First, only parents from 3 regions in Tanzania were included, and cultural differences between ethnic groups and communities may introduce diversity in the development and validation of this app. Tanzania has approximately 125 ethnic groups, and future studies could consider the inclusion of participants from other regions. Second, the study was carried out with no control group. Future studies should consider using a case-control model to eliminate potential confounders not evident in this study.

Additionally, although parents responded to the questionnaire immediately after using the game, there may be a disparity between the questionnaire response and parents’ behavior in real life when talking to their children. Moreover, measures of courtesy and recall bias were not performed in this study to determine if children could remember the materials. Further study would benefit from longitudinal evaluation of children’s behavior following use of the game. Finally, the COVID-19 pandemic has changed the world’s social ecosystem, including social restrictions and more indoor activities resulting in fewer interactions. The COVID-19 global pandemic also limited the total number and demographic of participants that could be included the study.

Parents are aware that they need to teach their children about sexual education, yet they are unaware of the most appropriate way to deliver this information. Hence, there is a need for game developers to design and validate different approaches to CSAPE based on the sociocultural differences of the target population. This study has contributed to the empirical discourse in designing an appropriate platform and media application against CSA using preventive games. HappyToto was designed in collaboration with game users, and was consequently able to incorporate the social cultural perspectives of the target community. Thus, the design and activities in the HappyToto game complement previous efforts in CSAPE in a culturally relevant way. This is important in ensuring that parents are comfortable with and adopt the use of CSAPE materials. There is a need for government and other appropriate authorities to support needed research on more attractive interactive features in teaching prevention and encouraging children to repeat the activities just enough to be able to remember the skills without becoming addicted to the game. Parents need to be informed that children as young as 3 years can be taught prevention education and can understand this education. This developed game will be further piloted in a wider population of children to assess its effectiveness. After the large-scale pilot and analysis of comments on the game design and content included in the game are completed, the game will be made available for free on the App Store and Google Play. Following this, we will develop a monitoring, assessment, and continuous improvement strategy, and increase awareness through schools and the media. Furthermore, we will perform technological demonstrations to private and governmental organizations that deal with children's welfare on a local and national level to promote awareness of CSAP and educate on how to use mobile technology to prevent CSA.

## Appendix

Multimedia Appendix 1 Questionnaire for parents and caretakers on child sexual abuse prevention education.

Multimedia Appendix 2 Questionnaire for child experts on child sexual abuse prevention education.

Multimedia Appendix 3 Questionnaire to parents and caretakers on the validation of the game as an educational tool for child sexual abuse prevention.

## Abbreviations

CSA: child sexual abuse
CSAP: child sexual abuse prevention
CSAPE: child sexual abuse prevention education
GDLC: game development life cycle
MoHCDGEC: Tanzania’s Ministry of Health, Community Development, Gender, Elderly and Children
UI: user interface.
