# Supplementary material for: Development and Validation of a Mobile Game for Culturally Sensitive Child Sexual Abuse Prevention Education in Tanzania: Mixed Methods Study
Source: JMIR Serious Games. 2021 Nov 8;9(4):e30350. doi: 10.2196/30350 (PMC8663517; doi:10.2196/30350)
Supplement: Multimedia Appendix 1 [file games_v9i4e30350_app1.pdf]

The Nelson Mandela
African Institution of Science
and Technology

CONSENT FORM FOR PARENT/CARETAKER/CHILD EXPERT TO PARTICIPATE IN RESEARCH

Read: Hi, My name is Maria Proches Malamsha from NM-AIST

You are invited to participate in this research and you need to know the aim before deciding whether to participate or not. Your participation in this research is of free will and it will not affect you in any way. The nature of questions was focused solely on application design and not on experiences so you can stop at any time when not comfortable. However there will be no payment of any sort for your participation. You can ask question for clarification if you do not understand something before you answer questions and you can also be give the copy of this form.

The aim of this research is using mobile technology to help parents and caretakers protect their children from Child sexual abuse in Tanzania. If you agree to answer this questionnaire, please put your signature in this form to confirm that you have agreed the terms and you will give full cooperation and correct answers.

Benefits of participating in this research is we believe that it will enable us to get more information about child sexual abuse and assist into designing application to help children protect themselves. There is no any drawback from the research. Records will be given unique identifiers that cannot be traced back to you and stored in a safe place where only researchers can have Access to.

WHO TO CONTACT?

If any concern arises from this research you can communicate with the researcher Ms. Maria Malamsha +255 725 261 702.

WOULD YOU LIKE TO PARTICIPATE? YES…………./**NO**…………..(if the answer is YES then you can proceed)

CERTIFICATE OF CONCENT

I have read the foregoing information, or it has been read to me. I have had the opportunity to ask questions about it and any questions that I have asked have been answered to my satisfaction. I consent voluntarily to participate in this study.

Name ________________________________

Signature (Finger print)__________________Date________________

The Nelson Mandela African Institution of Science and Technology

QUESTIONNAIRE TO PARENTS/CARETAKERS ON CHILD SEXUAL ABUSE EDUCATION

Welcome to this questionnaire.

The current questionnaire is being conducted as part of Masters in Information and communication science and engineering research at the Nelson Mandela African Institution of Science and Technology (NM-AIST), Tanzania aimed at developing a child mobile phone game Application for delivering sexual abuse prevention (CSA) education. The Application is intended to assist parents, care-givers and teachers in talking to children about sexual abuse and how can be prevented. The nature of questions was focused solely on application design and not on experiences so you can exit anytime when you do not feel comfortable. The privacy of all participants will be strictly ensured and any information provided will be used only for the purpose of this research.

A. GENERAL INFORMATION

s/no.		
1.	Region	
2.	District	
3.	Location (GPS if available)	
4.	Household Head Name (optional)	
5.	Sex of the HH	Male ( ) Female ( )
6.	Age of the HH *(please tick (√ where appropriate)*	Below 20 ( ) 20-30 ( ) 31-40 ( )<br>41-50 ( ) Above 50 ( )
7.	Household Head (HH) Marital Status *(please tick (√ where appropriate)*	Married ( ) Single ( )<br>Other (specify) ( )
8.	Name of the Interviewee (*if different from HH*, Optional)	
9.	Sex of the Interviewee(*tick the appropriate answers)*	Male ( ) Female ( )

10.	Age of the Interviewee(*tick the appropriate answers)*	Below 20 ( ) 20-30 ( ) 31-40 ( )<br>41-50 ( ) Above 50 ( )
11.	Occupation *(please tick (√ where appropriate)*	Farmer ( ) Business ( )<br>Salaried employment ( ) Self-employment ( )<br>Other (specify) ( )
12.	Education level of the Interviewee(*tick the appropriate answers)*	Primary level ( ) Secondary Level ( ) Tertiary level ( ) Other ( )
13.	How many children do you have? (*tick the appropriate answers)*	(i) 1 (ii) 2 (iii) 3 (iv) 4 (v) More than 4

B. SOCIAL CULTURAL PRACTICES

Social culture environment is set of believes, practices and behaviors that exists within a population.

14. How do children in your community including your learn about child abuse topics?
(*tick the appropriate answers)*

i. From parents ( )
ii. From Teachers ( )
iii. From aunties ( )
iv. Neighbor/society ( )
v. Other (Specify)………………………………………………………………………
………………………………………………………………………………..

15. Write True or False in the following

vi. Child sexual abuse is a worldwide crisis ( )
vii. Only girls can be sexually abused. ( )
viii. Child sexual abuse can be prevented through education. ( )
ix. When a child is abused it is their fault. ( )

16. When a child asks you about sexual abuse related topic what do you do?
(*tick the appropriate answers)*

i. Get anxiety ( )
ii. Ignore/ they will learn when they grow up ( )
iii. Send them to their teachers/religious teachers ( )
iv. Send them to auntie/another adult ( )
v. Warning them not to talk about such things ( )
vi. I will talk to them ( )

vii. Other ……………………………………………………………………

…………………………………………………………………………………

17. When can a child start learning about child sexual abuse? (*tick the appropriate answers)*
   i. 0-3 ( ) ii. 4-9 ( ) iii. 10-13 ( ) iv. 14-18 ( )

18. What are the barriers of you talking about CSA with your child? (*tick the appropriate answers)*
   i. Parents are unwilling/ children will know too much about sex ( )
   ii. Discomfort in discussing the topic ( )
   iii. Inadequate prevention knowledge and skills by parents ( )
   iv. It is not a big problem ( )
   v. no time/parents are no responsible ( )
   vi. Immoral activities (culture and religion wise) FGM ( )
   vii. Poverty ( )
   viii. Other specify……………………………………………………………

19. What are the enables of you talking about CSA with your child? (*tick the appropriate answers)*
   i. I want to protect my child ( )
   ii. The increase of abuse reports ( )
   iii. Globalization ( )
   iv. Other specify……………………………………………………………

C. DESIGNING OF THE APPLICATION

20. What kind of phone do you use? (*tick the appropriate answers)*
   Android ( )
   IOS ( )
   Windows mobile ( )
   Blackberry ( )

21. Have you ever played/downloaded a game app? (*tick the appropriate answers)*
   i. Yes ( ) ii. No ( )

22. How often do you play them? (*tick the appropriate answers)*
   i. Daily ii. Weekly iii. Monthly iv. Yearly

23. If a mobile application game for children ware to be developed, Which topics should be included? (*tick the appropriate answers)*
   i. Safety touches ( )
   ii. Correct names of private parts ( )
   iii. Growth and development ( )

iv. learning to say 'No' in response to a sexual abuse lure(gift) ( )
v. An abuser can be any person ( )
vi. You need to leave and report in risky situation ( )
vii. Ignoring bad myth that support (for parents) ( )
viii. Others (specify)……………………………………………………..

24. Which mode will you be comfortable for your child to learn about sexual abuse prevention using the application? (*tick the appropriate answers)*
   i. When they play alone ( )
   ii. When they play under your supervision ( )
   iii. Both ( )
   iv. Other……………………………………………………………….

25. What local games/songs do they use to teach children about sexual abuse prevention? Do they ensure retention of knowledge (say yes or no at the end of the name)?

…………………………………………………………………………………

…………………………………………………………………………………

…………………………………………………………………………………

26. Do you let your child play with games in your phone? (*tick the appropriate answers)*
   i. Yes ( ) ii. No ( )

27. What phone games does your child like to play? (*tick the appropriate answers)*
   i. Kinds songs ( )
   ii. Car games/avatar/fighting ( )
   iii. Learning games (1,2,3 or a,b,c) ( )
   iv. Bible/Quran games ( )
   v. Others ………………………………………………….
   ………………………………………………………….

28. What mode of play of the game would you prefer? (*tick the appropriate answers)*
   i. One and off activity ( )
   ii. Story line ( )
   iii. Other ( )

29. How should the game be designed to address the traditional and habitual challenges in our society? *(you can draw or narrate considering the topics) e.g names of pars of the body, the environment that surrounds the child.*

   i. At the beginning of the game (information about the abuse, ways to talk to children, testimonies, should have a storyline content)

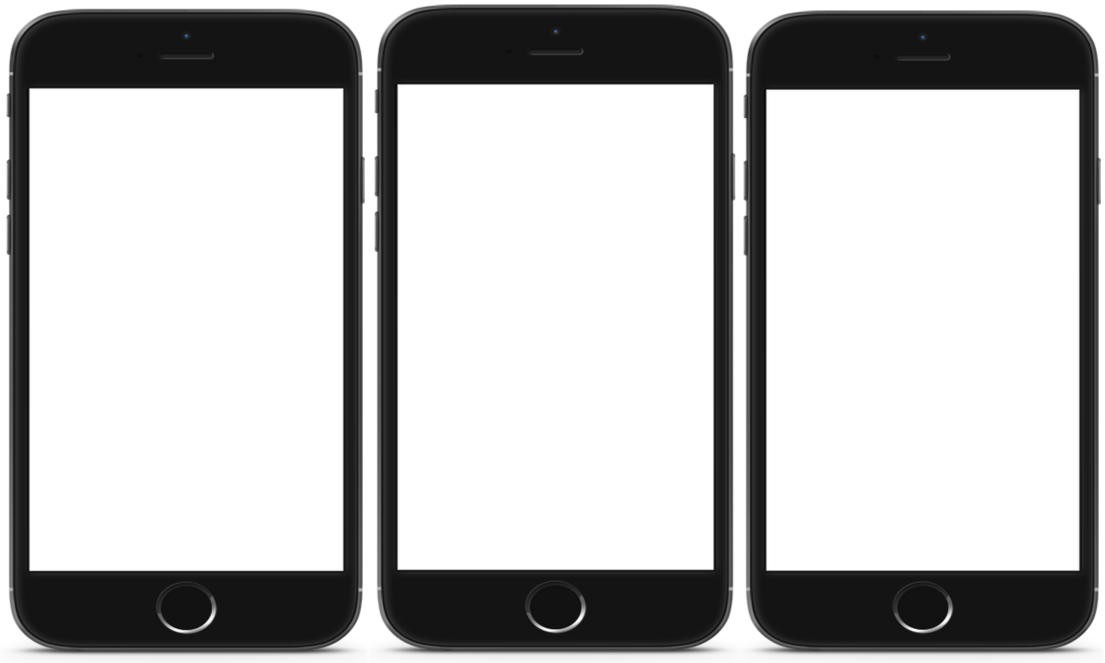

   ii. Important features that the game should not miss.(*mention them*)

………………………………………………………………………………………………..

……………………………………………………………………………………………….

……………………………………………………………………………………………….

……………………………………………………………………………………………….

……………………………………………………………………………………………….

………………………………………………………………………………………………

30. What applications do use/know of prevention science/getting more information?

   (tick the appropriate choice)

   i. Exercise application ( )
   ii. Pregnancy application (Women) ( )
   iii. Periods Application (Women) ( )
   iv. Other ……………………………………………………………………

31. Indicate your important features for the application. (*tick the appropriate answers)*

i. Replay ( )
ii. Cost effective ( )
iii. Interesting (colorful characters, interactivity) ( )
iv. Rewards ( )
v. Constant updates ( )

D. Ecological systems

It is a system of relationships that form his or her (child) environment that affect their development (personal relationship with family, classmates, teachers and care givers).

32. Which of the following ecological settings pose abuse threats to children? (*tick the appropriate answers)*

i. Single parent family ( )
ii. Brought up by relatives ( )
iii. Presence of step parents ( )
iv. Presence of alcoholism in the family ( )
v. Extended family ( )
vi. Foster care ( )
vii. Boarding schools ( )
viii. Other (specify)……………………………………………………………

………………………………………………………………………………

33. Do you normally check your child's body for any changes (abuse signs)? (*tick the appropriate answers)*

i. Yes ( ) ii. No ( )

34. If yes, how many times?(*tick the appropriate answers)*

i. daily ( ) ii. weekly( ) iii. monthly( ) iv. Rarely ( )
