# Supplementary material for: Development and Validation of a Mobile Game for Culturally Sensitive Child Sexual Abuse Prevention Education in Tanzania: Mixed Methods Study
Source: JMIR Serious Games. 2021 Nov 8;9(4):e30350. doi: 10.2196/30350 (PMC8663517; doi:10.2196/30350)
Supplement: Multimedia Appendix 2 [file games_v9i4e30350_app2.pdf]

The Nelson Mandela
African Institution of Science
and Technology

CONSENT FORM FOR PARENT/CARETAKER/CHILD EXPERT TO PARTICIPATE IN RESEARCH

Read: Hi, My name is Maria Proches Malamsha from NM-AIST

You are invited to participate in this research and you need to know the aim before deciding whether to participate or not. Your participation in this research is of free will and it will not affect you in any way. The nature of questions was focused solely on application design and not on experiences so you can stop at any time when not comfortable. However there will be no payment of any sort for your participation. You can ask question for clarification if you do not understand something before you answer questions and you can also be give the copy of this form.

The aim of this research is using mobile technology to help parents and caretakers protect their children from Child sexual abuse in Tanzania. If you agree to answer this questionnaire, please put your signature in this form to confirm that you have agreed the terms and you will give full cooperation and correct answers.

Benefits of participating in this research is we believe that it will enable us to get more information about child sexual abuse and assist into designing application to help children protect themselves. There is no any drawback from the research. Records will be given unique identifiers that cannot be traced back to you and stored in a safe place where only researchers can have Access to.

WHO TO CONTACT?
If any concern arises from this research you can communicate with the researcher Ms. Maria Malamsha +255 725 261 702.

WOULD YOU LIKE TO PARTICIPATE? YES…………./**NO**…………..(if the answer is YES then you can proceed)

CERTIFICATE OF CONCENT
I have read the foregoing information, or it has been read to me. I have had the opportunity to ask questions about it and any questions that I have asked have been answered to my satisfaction. I consent voluntarily to participate in this study.
Name ______________________________

Signature (Finger print)_________________Date_______________

The Nelson Mandela African Institution of Science and Technology

QUESTIONNAIRE FOR CHILD EXPERTS

Welcome to this interview.

The current interview is being conducted as part of Masters in Information and communication science and engineering research at the Nelson Mandela African Institution of Science and Technology (NM-AIST), Tanzania aimed at developing a child mobile phone game Application for delivering sexual abuse prevention (CSA) education. The Application is intended to assist parents, care-givers and teachers in talking to children about sexual abuse and how can be prevented. The nature of questions was focused solely on application design and not on experiences so you can exit anytime when you do not feel comfortable. The privacy of all participants will be strictly ensured and any information provided will be used only for the purpose of this research.

A. GENERAL INFORMATION

S/N		
1.	Region	
2.	District	
3.	Name (optional)	
4.	Age *(please tick (√ where appropriate)*	Below 20 ( ) 20-30 ( ) 31-40 ( )<br>41-50 ( ) Above 50 ( )
5.	Sex	Male ( ) Female ( )
6.	Occupation *(please tick (√ where appropriate)*	Social Worker( ) Teacher ( ) Police ( )<br>Child Psychologist ( )<br>Other (specify) ( )
7.	Education level (*tick the appropriate answers)*	Primary level ( ) Secondary Level ( ) Tertiary level ( ) Other ( )

B. SOCIAL CULTURAL PRACTICES:

Social culture environment is set of believes, practices and behaviors that exists within a population.

8. Is there a specific child sexual abuse prevention education policy in Tanzania?
i. Yes ( ) ii. No ( )
(if Yes, give reference)
…………………………………………………………………………………………

…………………………………………………………………………………………

…………………………………………………………………………………………

9. How is CSA education delivered in Tanzania and tick the most preferred? (open question)
   i. At family level…………………………………………………
   ....
   ii. At community level……………………………………………..
   …………………………………………………………………..
   iii. At school level………………………………………………….
   …………………………………………………………………..
   iv. Other related programs…………………………………………
   ……………………………………………………………………

10. When can a child start learning about child sexual abuse? (*tick the appropriate answers)*
i.0-3 ( ) ii. 4-9 ( ) iii. 10-13 ( ) iv. 14-18 ( )

11. What are the barriers of you talking about CSA with your child? (*tick the appropriate answers)*
   i. Parents are unwilling/ children will know too much about sex ( )
   ii. Discomfort in discussing the topic ( )
   iii. Inadequate prevention knowledge and skills by parents ( )
   iv. It is not a big problem ( )
   v. no time/parents are no responsible ( )
   vi. Immoral activities (culture and religion wise) FGM ( )
   vii. Poverty ( )
   viii. Other specify……………………………………………………

12. What are the enables of you talking about CSA with your child? (*tick the appropriate answers)*
   i. I want to protect my child ( )
   ii. The increase of abuse reports ( )
   iii. Globalization ( )
   iv. Other specify………………………………………………

C. DESIGNING OF THE APPLICATION

13. What kind of phone do you use? (*tick the appropriate answers)*

Android	( )
IOS	( )
Windows mobile	( )
Blackberry	( )

14. Have you ever played/downloaded a game app? (*tick the appropriate answers)*
    i. Yes ( ) ii. No ( )

15. How often do you play them? (*tick the appropriate answers)*
    i. Daily ii. Weekly iii. Monthly iv. Yearly
16. Do children play mobile phone games? i. Yes ( ) ii. No ( )

17. If a mobile application is to be developed to help parents get involved in the CSA education with their children, which topics should be included? (*Please tick most preferred topics in CSA education in Tanzania*)
    i. Safety touches ( )
    ii. Correct names of private parts ( )
    iii. Growth and development ( )
    iv. Don't receive gifts ( )
    v. An abuser can be any person ( )
    vi. You need to report ( )
    vii. Need for self-respect ( )
    viii. leaving the situation ( )
    ix. learning to say 'No' in response to a sexual abuse lure ( )
    x. Ignoring bad myth that support (for parents) ( )
    xi. Others (specify)………………………………………………………..

18. Which mode will you be comfortable for your child to learn about sexual abuse prevention using the application? (*tick the appropriate answers)*
    i. When they play alone ( )
    ii. When they play under your supervision ( )
    iii. Both ( )
    iv. Other……………………………………………………………………

19. What local games/songs do they use to teach children about sexual abuse prevention? Do they ensure retention of knowledge (say yes or no at the end of the name)?

………………………………………………………………………………………

………………………………………………………………………………………

20. What mode of play of the game would you prefer? (*tick the appropriate answers)*

    i. One and off activity ( )
    ii. Story line ( )
    iii. Other ( )

21. How should the game be designed to address the traditional and habitual challenges in our society? *(open question, you can draw or narrate)*

    i. At the beginning of the game

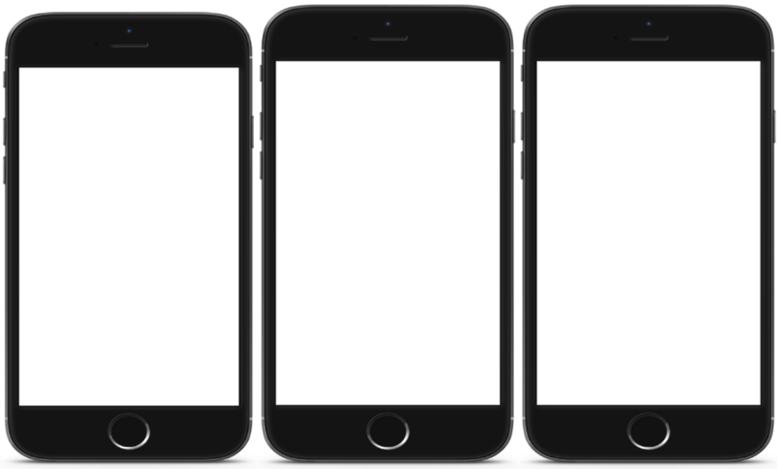

    ii. Important features that the game should not miss.(mention them)
    …………………………………………………………………………
    …………………………………………………………………………..
    …………………………………………………………………………
    …………………………………………………………………………..
    …………………………………………………………………………...

22. Aware of mobile applications used for prevention education?
    If Yes please tick the appropriate choice.

    i. HIV application ( )
    ii. Pregnancy application (Women) ( )
    iii. Periods Application (Women) ( )
    iv. Games for children ( )
    v. Other …………………………………………………………………………

    ………………………………………………………………………………….

13. ECOLOGICAL SYSTEMS

It is a system of relationships (personal relationship with family, classmates, teachers and care givers) within the children environment that affects their development.

23. What are the environmental conditions you think should be considered in the child sexual abuse prevention education? (*tick the appropriate answer*)

i.	Single parent family	( )
ii.	Brought up by relatives	( )
iii.	Presence of step parents	( )
iv.	Presence of alcoholism in the family	( )
v.	Poverty	( )
vi.	Extended family	( )
vii.	Foster care	( )
viii.	Boarding schools	( )
ix.	other (specify)…………………………………………………………………	

…………………………………………………………………………………

24. Do you normally check your child's body for any changes (abuse signs)? (*tick the appropriate answers)*

i. Yes ( ) ii. No ( )

25. If yes, how many times and at what time?,(*tick the appropriate answers)*

i. daily ( ) ii. weekly( ) iii. monthly( ) iv. Rarely ( )
