# Supplementary material for: Development and Validation of a Mobile Game for Culturally Sensitive Child Sexual Abuse Prevention Education in Tanzania: Mixed Methods Study
Source: JMIR Serious Games. 2021 Nov 8;9(4):e30350. doi: 10.2196/30350 (PMC8663517; doi:10.2196/30350)
Supplement: Multimedia Appendix 3 [file games_v9i4e30350_app3.docx]

**CONSENT FORM FOR PARENT/CARETAKER**

**TO PARTICIPATE IN RESEARCH**

**Read:** Hi, My name is Maria Proches Malamsha from NM-AIST

You are invited to participate in this research and you need to know the aim before deciding whether to participate or not. Your participation in this research is of free will and it will not affect you in any way. The nature of questions was focused solely on application design and not on experiences so you can stop at any time when not comfortable. However there will be no payment of any sort for your participation. You can ask question for clarification if you do not understand something before you answer questions and you can also be give the copy of this form.

The aim of this research is using mobile technology to help parents and caretakers protect their children from Child sexual abuse in Tanzania. If you agree to answer this questionnaire, please put your signature in this form to confirm that you have agreed the terms and you will give full cooperation and correct answers.

Benefits of participating in this research is we believe that it will enable us to get more information about child sexual abuse and assist into designing application to help children protect themselves. There is no any drawback from the research. Records will be given unique identifiers that cannot be traced back to you and stored in a safe place where only researchers can have Access to.

**WHO TO CONTACT?**

If any concern arises from this research you can communicate with the researcher Ms. Maria Malamsha +255 725 261 702.

**WOULD YOU LIKE TO PARTICIPATE? YES**…………./**NO**…………..(if the answer is YES then you can proceed)

**CERTIFICATE OF CONCENT**

I have read the foregoing information, or it has been read to me. I have had the opportunity to ask questions about it and any questions that I have asked have been answered to my satisfaction. I consent voluntarily to participate in this study.

Name _______________________________

Signature (Finger print)__________________Date________________

**QUESTIONNAIRE TO PARENTS/CARETAKERS ON VALIDATION OF GAME AS AN EDUCATIONAL TOOL FOR CHILD SEXUAL ABUSE**

**Welcome to this questionnaire**

The current questionnaire is being conducted as part of Masters in Information and communication science and engineering research at the Nelson Mandela African Institution of Science and Technology (NM-AIST), Tanzania aimed at developing a child mobile phone game Application for delivering sexual abuse prevention (CSA) education. The Application is intended to assist parents, care-givers and teachers in talking to children about sexual abuse and how can be prevented. The privacy of all participants will be strictly ensured and any information provided will be used only for the purpose of this research.

1. **GENERAL INFORMATION**

| s/no. |  | |
| --- | --- | --- |
|  | Participant |  |
|  | Group number |  |
|  | Sex of the Interviewee(*tick the appropriate answers)* | Male ( ) Female ( ) |
|  | Age of the Interviewee(*tick the appropriate answers)* | Below 20 ( ) 20-30 ( ) 31-40 ( )  41-50 ( ) Above 50 ( ) |
|  | Interviewee (HH) Marital Status *(please tick (* **√** *where appropriate)* | Married ( ) Single ( )  Other (specify) ( ) |
|  | Occupation *(please tick (* **√** *where appropriate)* | Farmer ( ) Business ( )  Salaried employment ( ) Self-employment ( )  Other (specify) ( ) |
|  | Education level of the Interviewee(*tick the appropriate answers)* | Primary level ( ) Secondary Level ( ) Tertiary level ( ) Other ( ) |
|  | How many children do you have? (*tick the appropriate answers)* | (i) 1 (ii) 2 (iii) 3 (iv) 4 (v) More than 4 |

1. **BEFORE USING THE APPLICATION**

| No | Question |  | | | | |
| --- | --- | --- | --- | --- | --- | --- |
| 1 | How much is your child (3-5 years) capable of ? | Not at all | Somehow | Neutral | Good | Very Good |
|  | 1. Listening and taking up instructions | 1 | 2 | 3 | 4 | 5 |
|  | 1. Reading | 1 | 2 | 3 | 4 | 5 |
|  | 1. Understanding images and video | 1 | 2 | 3 | 4 | 5 |
| 2 | How comfortable are you in teaching your child about these topics, | Uncomfortable | Somehow  Uncomfortable | Neutral | Somehow  Comfortable | Comfortable |
|  | 1. Types of private parts in the body   Reasons for your choice/answer........................................................  ………………………  . | 1 | 2 | 3 | 4 | 5 |
|  | 1. When and from who to receive presents?   Reasons for your choice/answer.......................................................  ………………………………………………. | 1 | 2 | 3 | 4 | 5 |
|  | 1. what group of people should the child avoid/be careful of, and what to do in dangerous environments and when someone exhibits a dangerous behavior?   Reasons for your choice/answer........................................................  ………………………..……………………….. | 1 | 2 | 3 | 4 | 5 |

1. How would you teach your child about the following topics?

1. Types of private parts in the body

…………………………………………………………………………………………..

…………………………………………………………………………………………..

1. Where and from who to receive presents

…………………………………………………………………………………………..

…………………………………………………………………………………………..

1. what group of people should the child avoid/be careful of, and what to do in dangerous environments and when someone exhibits a dangerous behavior?

…………………………………………………………………………………………..

…………………………………………………………………………………………..

1. In the course of protecting your child/children against sexual abuse what would you want your child/children to learn (how much information should they get):-
2. Types of private parts in the body

…………………………………………………………………………………………..

…………………………………………………………………………………………..

1. Where and from who to receive presents

…………………………………………………………………………………………..

…………………………………………………………………………………………..

1. what group of people should the child avoid/be careful of, and what to do in dangerous environments and when someone exhibits a dangerous behavior?

…………………………………………………………………………………………..

…………………………………………………………………………………………..

1. **ACCEPTABILITY OF THE APPLICATION (AFTER PARENTS HAVE USED THE APP)**

| No | Question*(Tick the appropriate answer)* |  | | | | |
| --- | --- | --- | --- | --- | --- | --- |
| 1 | How comfortable are you in teaching your children about, | Uncomfortable | Somehow  uncomfortable | Neutral | Somehow  comfortable | comfortable |
|  | 1. Types of private parts in the body | 1 | 2 | 3 | 4 | 5 |
|  | 1. Where and from who to receive presents? | 1 | 2 | 3 | 4 | 5 |
|  | 1. what group of people should the child avoid/be careful of…and what to do in dangerous environments and when someone exhibits a dangerous behavior? | 1 | 2 | 3 | 4 | 5 |
| 2 | Do you think the application conforms with the Tanzanian culture? (topics, language, environment) | Inappropriate | Somehow  Inappropriate | Neutral | Somehow  Appropriate | Appropriate |
|  | 1. Types of private parts in the body   Reasons for your choice/answer ……………………………  …………………………… | 1 | 2 | 3 | 4 | 5 |
|  | 1. When and from who to receive presents?   Reasons for your choice/answer ……………………………………………………………………………… | 1 | 2 | 3 | 4 | 5 |
|  | 1. what group of people should the child avoid/be careful of…and what to do in dangerous environments and when someone exhibits a dangerous behavior?   Reasons for your choice/answer ……………………………………………………….................................... | 1 | 2 | 3 | 4 | 5 |
| 3 | How would you rate your overall satisfaction with this app as an education tool (message, graphics)  Reasons for your choice/answer  ……………………………………………………………………………………………… | dissatisfied | Somehow  Dissatisfied | Neutral | Somehow  Satisfied | Satisfied |
| 4 | Do you think Child’s innocence is affected with the game? (Do you think a child will be anxious after using the game?)  Reasons for your choice/answer  ………………………………………………………………………… | Very much | Somehow affected | Neutral | Somehow  Not affected | Not affected |

1. How would you teach your child about the following topics?
2. Types of private parts in the body

…………………………………………………………………………………………..

…………………………………………………………………………………………..

1. Where and from who to receive presents

…………………………………………………………………………………………..

…………………………………………………………………………………………..

1. what group of people should the child avoid/be careful of, and what to do in dangerous environments and when someone exhibits a dangerous behavior?

…………………………………………………………………………………………..

…………………………………………………………………………………………..

1. Will you allow your child to play the game? i. Yes ii. No

Reasons for your choice/answer

…………………………………………………………………………………………..

………………………………………………………………………………………….

1. Will you recommend this game to other parents? i. Yes ii. No

Reasons for your choice/answer

………………………………………………………………………………………….

……………………………………………………………………………………….

1. What features did you like the most in the game and Reasons for your choice/answer

………………………………………………………………………………………..

……………………………………………………………………………………….

………………………………………………………………………………………..

1. What features did you like the least and Reasons for your choice/answer

………………………………………………………………………………………..

……………………………………………………………………………………….

………………………………………………………………………………………..

1. What topics/ features should be added? Reasons for your choice/answer

………………………………………………………………………………………..

……………………………………………………………………………………….

………………………………………………………………………………………..

1. What do you consider as safe network for your child? *(Tick the appropriate answer)*

i. Father ii. Mother iii. Sisters iv. Brothers v. Aunties vi. Uncles vii. Neighbors

viii. Teachers at school ix. Others(specify)……………………………………

1. **USABILITY OF THE APPLICATION**

| No | Question *(Tick the appropriate answer)* | Disagree | Somehow  Disagree | Neutral | Somehow  Agree | Agree |
| --- | --- | --- | --- | --- | --- | --- |
| 1 | I think that I would like my child to play this game frequently. | 1 | 2 | 3 | 4 | 5 |
| 2 | I found the game unnecessary for my child | 1 | 2 | 3 | 4 | 5 |
| 3 | I thought the game was easy to play and I could navigate to all/many parts. | 1 | 2 | 3 | 4 | 5 |
| 4 | I think that my child will need support to play this game. | 1 | 2 | 3 | 4 | 5 |
| 5 | I found the game activities in this game well integrated. | 1 | 2 | 3 | 4 | 5 |
| 6 | I thought there was too much inconsistency in this game. | 1 | 2 | 3 | 4 | 5 |
| 7 | I would imagine that most children love animation so would learn to use this game very quickly. | 1 | 2 | 3 | 4 | 5 |
| 8 | I found the animation hard for children to understand. | 1 | 2 | 3 | 4 | 5 |
| 9 | I felt very confident using the game. | 1 | 2 | 3 | 4 | 5 |
| 10 | I needed to learn a lot of things before I could get going with this | 1 | 2 | 3 | 4 | 5 |

1. **ACCEPTABILITY OF THE APPLICATION (CHILDREN)**
2. Did you like playing the game? i. Yes ii. No

Reasons for your choice/answer

…………………………………………………………………………………………

1. What did you like in the game?

Reasons for your choice/answer

……………………………………………………………………………………­­­

1. What do you if a neighbor tells you to take off your clothes?

………………………………………………………………………………………….

1. What do you if someone on the way gives you biscuit?

…………………………………………………………………………………………..

1. If uncle calls you in his room at home what will you do?

…………………………………………………………………………………………..
